# PRDM14 Is a Unique Epigenetic Regulator Stabilizing Transcriptional Networks for Pluripotency

**DOI:** 10.3389/fcell.2018.00012

**Published:** 2018-02-13

**Authors:** Yoshiyuki Seki

**Affiliations:** Department of Biomedical Chemistry, School of Science and Technology, Kwansei Gakuin University, Hyogo, Japan

**Keywords:** Prdm14, epigenetics, embryonic stem cells (ESCs), primordial germ cells, DNA Methylation

## Abstract

PR-domain containing protein 14 (PRDM14) is a site-specific DNA-binding protein and is required for establishment of pluripotency in embryonic stem cells (ESCs) and primordial germ cells (PGCs) in mice. DNA methylation status is regulated by the balance between *de novo* methylation and passive/active demethylation, and global DNA hypomethylation is closely associated with cellular pluripotency and totipotency. PRDM14 ensures hypomethylation in mouse ESCs and PGCs through two distinct layers, transcriptional repression of the DNA methyltransferases *Dnmt3a/b/l* and active demethylation by recruitment of TET proteins. However, the function of PRDM14 remains unclear in other species including humans. Hence, here we focus on the unique characteristics of mouse PRDM14 in the epigenetic regulation of pluripotent cells and primordial germ cells. In addition, we discuss the expression regulation and function of PRDM14 in other species compared with those in mice.

## Role of PRDM14 in DNA demethylation

PRDM14 functions as a site-specific transcriptional activator and repressor through the recruitment of transcriptional regulators in mouse ESCs (mESCs) (Yamaji et al., 2008; Chia et al., 2010; Ma et al., 2011). *Prdm14* is transiently expressed in the inner cell mass (ICM) of the blastocyst, followed by rapid downregulation in the epiblast at the post-implantation stage in mice (Yamaji et al., 2008). Primordial germ cells (PGCs) are specified from the most proximal and posterior epiblast cells through BMP4 signaling approximately at embryonic day (E) 6.25 (Lawson et al., 1999). Nascent PGCs enter the embryo proper approximately at E8.0 and migrate through the hindgut, eventually colonizing the embryonic gonad, which constitutes the future testis or ovary. *Prdm14* is specifically upregulated during PGC specification from the epiblast and is required for early development of PGCs (Yamaji et al., 2008). In developing PGCs, DNA demethylation occurs in a stepwise manner at the migrating and arriving phases (Seki et al., 2005). DNA demethylation is regulated by two pathways: replication-dependent mechanisms and replication-independent mechanisms (Wu and Zhang, 2017). Soon after PGC specification, the essential factors for epigenomic imprinting, *Dnmt3a, Dnmt3b*, and *Uhrf1*, are downregulated (Seisenberger et al., 2012; Kagiwada et al., 2013; Ohno et al., 2013; Kawasaki et al., 2014), which contributes to global DNA hypomethylation in developing PGCs. *Prdm14*-deficient PGCs fail to repress *Dnmt3b* and *Uhrf1* expression, thereby resulting in stalling of global DNA demethylation (Shirane et al., 2016).

**Figure 1:**
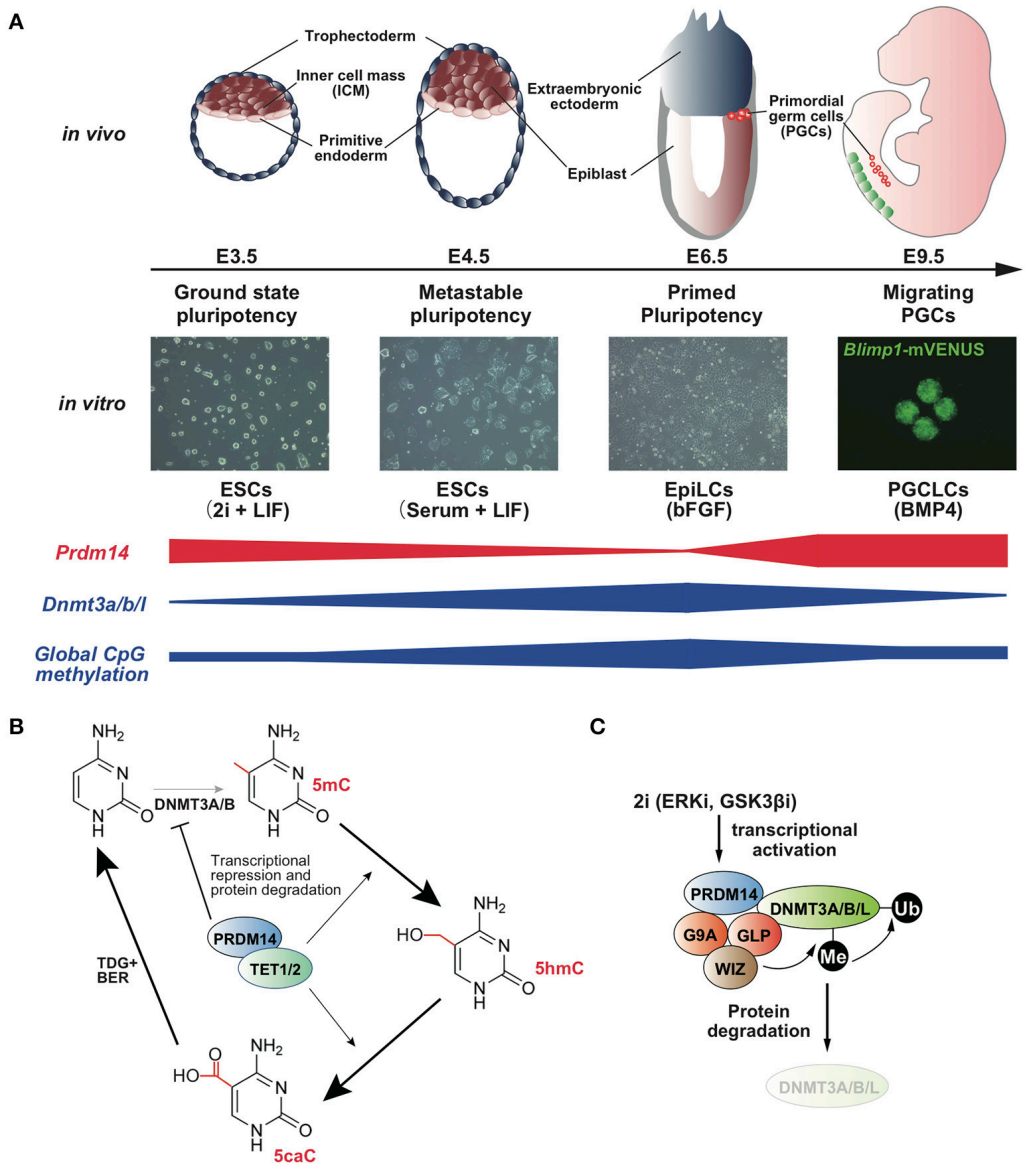
**(A)** *Prdm14* expression negatively correlates with global CpG methylation level in the states of pluripotency and PGC development (Yamaji et al., 2008, 2013). **(B)** Model for the acceleration of TET-TDG-BER-mediated DNA demethylation by PRDM14 (Okashita et al., 2014). **(C)** Model for protein degradation of DNMT3A/B/L by PRDM14 (Sim et al., 2017).

**Figure 2:**
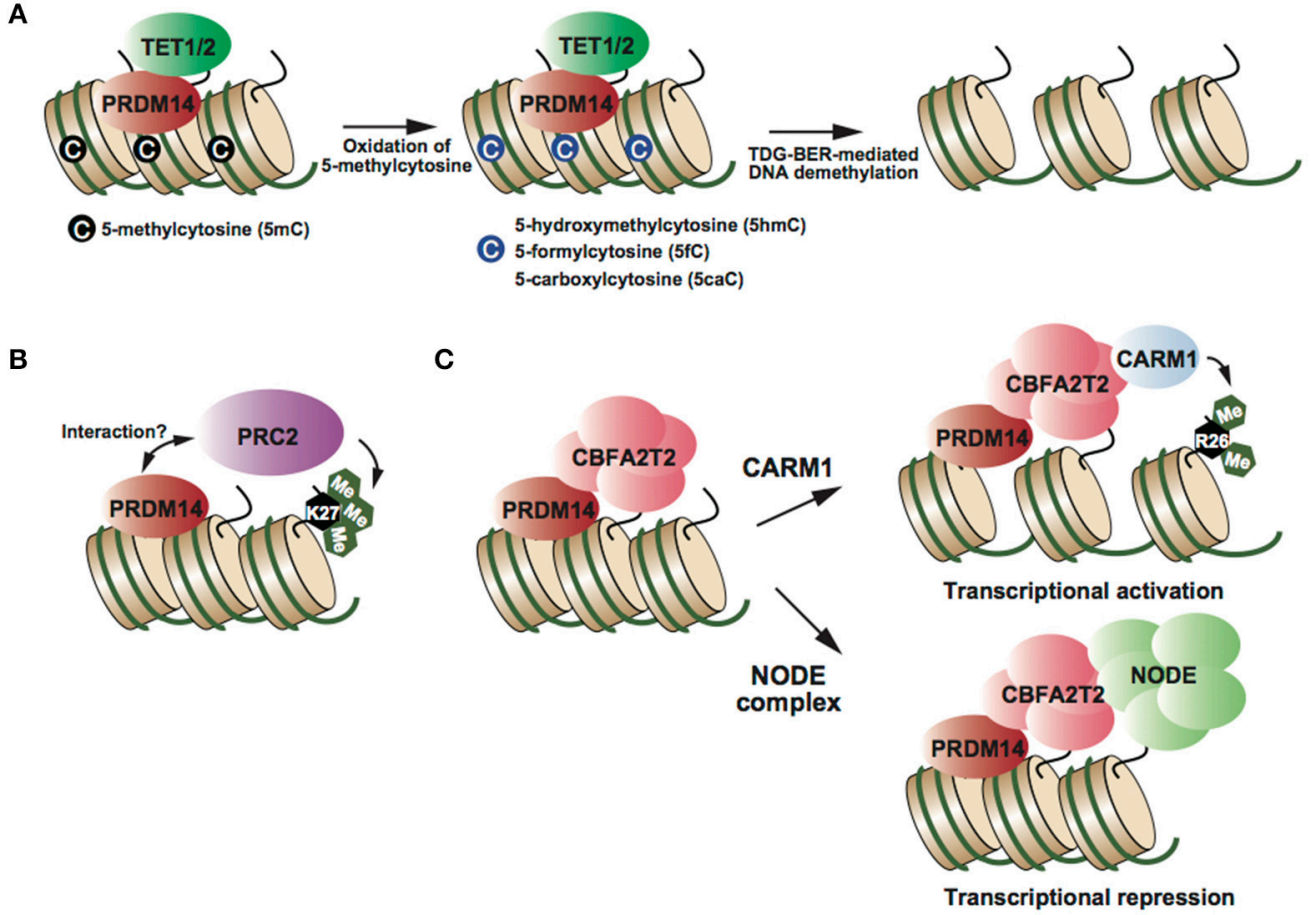
**(A)** PRDM14 recruits TET proteins at target genes, resulting in oxidation of 5mC and TDG-BER-mediated demethylation (Okashita et al., 2014). **(B)** PRDM14 might interact with PRC2 and its complex deposits H3K27me3, resulting in transcriptional repression (Yamaji et al., 2013). **(C)** CBFA2T2 is an essential partner of PRDM14 in both transcriptional activation and repression (Nady et al., 2015; Tu et al., 2016). Partner switching of the PRDM14-CBFA2T2 complex depending on target genes (Burton et al., 2013; Nady et al., 2015).

Mouse embryonic stem cells (mESCs) retain metastable pluripotency in serum containing leukemia inhibitory factor (LIF), whereas the transfer of culture conditions from serum plus LIF to the presence of two pharmacological inhibitors for ERK and GSK3β, called 2i plus LIF, leads to ground-state pluripotency associated with pronounced reduction in genome-wide DNA demethylation (Figure 1A) (Ying et al., 2008; Leitch et al., 2013). The resulting global hypomethylation status ensures the activation of pluripotency-associated genes and germline-specific genes. In the 2i plus LIF condition, *Dnmt3a/b/l* is downregulated, whereas *Prdm14* is upregulated (Leitch et al., 2013). Furthermore, DNA methylation levels and *Dnmt3a/b/l* expression are consistently maintained at high levels in *Prdm14*-deficient ESCs even in the presence of 2i plus LIF, and PRDM14 directly binds to the upstream region of *Dnmt3a/b/l* and represses the transcription of these genes (Yamaji et al., 2013; Okashita et al., 2014). These findings indicate that PRDM14 is responsible for global hypomethylation through transcriptional repression of *Dnmt3a/b/l* in ground-state ESCs (Leitch et al., 2013). A recent study has shown that PRDM14 forms a complex with G9a, a histone methyltransferase, and this complex degrades DNMT3A/B proteins via lysine methylation-dependent polyubiquitination (Figure 1C) (Sim et al., 2017). Together, transcriptional repression of *Dnmt3a/b/l* and DNMT3A/B/L degradation via PRDM14, along with rapid proliferation, leads to global DNA hypomethylation in ground-state ESCs. Furthermore, DNA methylation of pluripotency-associated genes, germline-specific genes, and imprinted loci was rapidly diminished by *Prdm14* induction through the ten-eleven translocation (TET)-thymine DNA glycosylase (TDG)-base excision repair (BER) pathway in ESCs with serum containing LIF (Figures 1B, 2A) (Okashita et al., 2014). Thus, PRDM14 regulates two parallel pathways, DNMT3A/B/L repression and TET recruitment at target loci, to ensure global DNA hypomethylation in ground-state ESCs and PGCs in mice.

## Role of the PRDM14 complex in transcriptional regulation

PRDM14 has multiple functions in transcriptional regulation, e.g., DNA demethylation and transcriptional activation and repression, depending on the target genes. Biochemical studies have identified CBFA2T2, NODE complex, and BRG1 complex as PRDM14-containing complexes in mESCs (Figure 2) (Nady et al., 2015; Tu et al., 2016). PRDM14 interacts with CBFA2T2 stoichiometrically; this interaction is required to maintain pluripotency in mESCs and early differentiation of PGCs (Nady et al., 2015; Tu et al., 2016). Comparison of global gene expression patterns between *Prdm14* knockout (KO) and *Cbfa2t2* KO ESCs, revealed that CBFA2T2 was involved in both transcriptional activation and repression via PRDM14 in mESCs (Tu et al., 2016). Therefore, the switching mechanism in transcriptional regulation by PRDM14 complexes might be controlled by another component of the PRDM14 complex. Some studies have reported that PRDM14 interacts with the polycomb repressor complex 2 (PRC2) (Figure 2B) (Payer et al., 2013; Yamaji et al., 2013), whereas other studies failed to reproduce this finding (Nady et al., 2015; Tu et al., 2016). Although PRC2 and H3K27me3 enrichment at PRDM14-binding regions, including *Dnmt3b* and *Xist* loci, decreases in *Prdm14*-deficient mESCs compared with that in wild-type ESCs (Payer et al., 2013; Yamaji et al., 2013), it is largely unknown whether PRDM14 directly recruits the PRC2 complex at these regions. PRDM14 has also been shown to interact with CARM1, a histone arginine methyltransferase (H3R26me2), in mESCs (Figure 2C) (Burton et al., 2013). *Prdm14* is expressed heterogeneously at the 4-cell stage, and PRDM14 overexpression in one blastomere at the 2-cell stage drives the predominant contribution of the inner cell mass (ICM) of the blastocyst, similar to the phenotype of CARM1 overexpression. Several studies have reported the functional correlation between PRDM14 and its binding partners; however, the direct functional interaction of PRDM14 with its binding partners in transcriptional regulation has not been investigated.

## Diverse functions and PRDM14 expression pattern in mice and humans

mESC self-renewal is maintained by the LIF signal, whereas self-renewal of human embryonic stem cells (hESCs) is sustained through bFGF and ACTIVIN signaling. Epiblast stem cells (EpiSCs) derived from epiblast cells at the post-implantation stage of mouse embryos can be expanded and maintained through bFGF and ACTIVIN signaling (Tesar et al., 2007). Therefore, hESCs and EpiSCs are considered to display common features corresponding with the post-implantation epiblast. The expression level of the genes associated with naïve pluripotency including *Esrrb, Tcl1*, and *Klf4* is lower in hESCs and EpiSCs than in mESCs (Sasaki et al., 2015). Recent single-cell transcriptome analyses comparing human and mouse epiblasts derived from the same stage of the developing embryo showed distinct gene expression profiles of epiblasts in humans and mice (Blakeley et al., 2015). Interestingly, *Prdm14* expression is closely associated with naïve pluripotency in mESCs and *Prdm14* is not expressed in EpiSCs, whereas hESCs showing primed-state pluripotency express *PRDM14*, and this expression is required for the maintenance of hESC pluripotency but not for EpiSC pluripotency (Chia et al., 2010). Furthermore, single-cell transcriptome analysis of pre- and post-implantation epiblasts in monkeys showed that *Prdm14* expression is retained in epiblast cells until soon before gastrulation (Nakamura et al., 2016). In the case of mice, the gene expression profile of epiblast cells dramatically changes after implantation, and it includes the downregulation of naïve pluripotency markers such as *Prdm14, Nanog*, and *Sox2* (Kurimoto et al., 2008). In contrast to mice, however, the post-implantation epiblast relatively retains its gene expression profile for several days, suggesting that primate epiblasts persist for self-renewal of pluripotency (Nakamura et al., 2016). Sustainable *Prdm14* expression results in self-renewal of mESCs and hESCs during embryoid body formation (Tsuneyoshi et al., 2008; Okashita et al., 2015). Furthermore, induction of *Prdm14* expression in epiblast-like cells (EpiLCs) differentiated from mESCs induces the conversion of EpiLCs into mESCs through activation of pluripotent markers and the repression of differentiation markers (Okashita et al., 2016). These findings clearly indicate that PRDM14 stabilizes the transcriptional network for pluripotency, and that PRDM14 downregulation is necessary for exit from pluripotency. Therefore, the elucidation of distinct regulatory mechanisms for *Prdm14* expression in humans and mice is an essential challenge to understand the differences in epiblast maturation between humans and mice.

Mouse and rat embryos display unique epiblast morphology, referred to as an egg cylinder, whereas epiblasts form discs in non-rodent mammals. Such morphological differences in epiblasts between the embryonic disc and egg cylinder correlate with the differences in gene expression profiles and morphology of embryonic stem cells. *Lagostomus maximus* (plains vizcacha), which is a fossorial rodent, develops flat embryonic discs, as observed in non-rodent mammals (Leopardo and Vitullo, 2017). SOX17 is a critical determinant of germ cell generation in humans but not in mice, and it is not expressed in mouse PGCs. Interestingly, primordial germ cells of *Lagostomus maximus* embryo express SOX17, as observed in human PGCs. These findings suggest that the embryonic disc constitutes the ancestral morphology of epiblasts in the common ancestor of rodents and muroidea including mice, rats, and hamsters, and might have acquired muroidea-specific mechanisms to change epiblast morphology during evolution from the rodent common ancestor.

## Functional and expression diversity of PRDM14 in deuterostomes

*Prdm14* is widely distributed in metazoans but not in ecdysozoans including *Drosophila* and *Caenorhabditis elegans* (Vervoort et al., 2016). However, limited information is available regarding the expression pattern and function of PRDM14 in non-mammalian deuterostomes. *Prdm14* is expressed in pluripotent cells and primordial germ cells in mammals; in contrast, *Prdm14* is expressed in motor neurons but not in primordial germ cells in the zebrafish embryo (Liu et al., 2012). In *Prdm14*-knock down zebrafish embryos, *islet2*, a critical transcription factor for motor neuron development, is downregulated in motor neurons, and PRM14 binds to the *islet2* locus, indicating that PRDM14 regulates motor neuron maturation through islet2 activation. It is unclear whether PRDM14 function in motor neurons is conserved among non-mammalian deuterostomes; therefore, the expression dynamics of *Prdm14* in non-mammalian deuterostomes warrant further investigation. Interestingly, *isl2*, a mouse homolog of zebrafish *islet2*, is also upregulated soon after *Prdm14* upregulation in mouse PGCs (Kurimoto et al., 2008), suggesting that the PRDM14-ISL2 axis might be co-opted from motor neurons to PGCs.

## Conclusion and further perspectives

Findings regarding the role of *Prdm14* expression in mouse germ cell specification led to the consideration that *Prdm14* is a critical determinant of germ cell fate in mammals. However, because the expression pattern of *Prdm14* differs among deuterostomes, the function of PRDM14 in deuterostomes could be reconsidered. I believe that this diversity of *Prdm14* expression depends on the mode of germ cell specification. Germ cell specification comprises two modes: preformation and epigenesis. During preformation, germ cell determinants are asymmetrically distributed in the oocyte, referred to as the “germplasm,” whereas germ cells are induced from pluripotent cells receiving extrinsic signals through epigenesis. It has been proposed that the early segregation of germ cells from somatic lineages might drive the diversity of somatic cells (Johnson and Alberio, 2015). Classical “model” organisms including zebrafish, *Xenopus*, and chicken employ preformation and early germ cell restriction mediated by BLIMP1, and this the phenomenon, referred to as “Blimping,” even takes place in mice, which undergo epigenesis. Therefore, comparison of the expression and function of *Prdm14* in organisms in which germ cell specification occurs in the late stage including urodele amphibians (axolotl), reptiles (gecko), and humans is necessary to uncover the evolutionarily conserved and diverse function of PRDM14 in pluripotent cells and primordial germ cells in deuterostomes.

## Author contributions

The author confirms being the sole contributor of this work and approved it for publication.

### Conflict of interest statement

The author declares that the research was conducted in the absence of any commercial or financial relationships that could be construed as a potential conflict of interest.
